# Disentangling Multiannual Air Quality Profiles Aided by Self-Organizing Map and Positive Matrix Factorization

**DOI:** 10.3390/toxics13020137

**Published:** 2025-02-14

**Authors:** Stefano Fornasaro, Aleksander Astel, Pierluigi Barbieri, Sabina Licen

**Affiliations:** 1 Department of Chemical and Pharmaceutical Sciences, University of Trieste, Via Giorgieri 1, 34127 Trieste, Italy; sfornasaro@units.it (S.F.); barbierp@units.it (P.B.); 2 Department of Environmental Chemistry and Toxicology, Pomeranian University in Słupsk, 22a Arciszewskiego Str., 76-200 Słupsk, Poland

**Keywords:** pollution, ambient air, particulate matter, NOx, multivariate analysis, self-organizing map, hierarchical clustering, positive matrix factorization, COVID-19

## Abstract

The evaluation of air pollution is a critical concern due to its potential severe impacts on human health. Currently, vast quantities of data are collected at high frequencies, and researchers must navigate multiannual, multisite datasets trying to identify possible pollutant sources while addressing the presence of noise and sparse missing data. To address this challenge, multivariate data analysis is widely used with an increasing interest in neural networks and deep learning networks along with well-established chemometrics methods and receptor models. Here, we report a combined approach involving the Self-Organizing Map (SOM) algorithm, Hierarchical Clustering Analysis (HCA), and Positive Matrix Factorization (PMF) to disentangle multiannual, multisite data in a single elaboration without previously separating the sites and years. The approach proved to be valid, allowing us to detect the site peculiarities in terms of pollutant sources, the variation in pollutant profiles during years and the outliers, affording a reliable interpretation.

## 1. Introduction

Air pollution assessment is a fundamental issue because air quality can have serious consequences for human health [1,2,3]. Nowadays, a huge amount of data are recorded at high frequency and, when trying to extract useful information, the researcher needs to cope with multiannual, multisite data as well as the possible presence of noise and sparse missing data [4,5].

Multivariate data analysis is widely used to face this challenge: beside well-established chemometrics such as Principal Component Analysis (PCA), Hierarchical Clustering Analysis (HCA), and k-means clustering (KM), there is a growing interest in neural networks and deep learning networks used for both analysis and prediction [5,6]. In this context, the Self-Organizing map algorithm has been successfully used for unsupervised analysis of large datasets [7,8,9,10]. The SOM algorithm is able to deal with large datasets and non-linear relationships among the variables and it is not significantly affected by outliers [11,12,13].

Another aim of air quality studies is to identify possible pollutant sources. In this context, the so-called receptor models are used [14,15]. Among them, Positive Matrix Factorization is widely used [16]. Nevertheless, there are few studies that use both SOM and PMF for environmental quality characterization, and they are mainly focused on water [17,18] and soil/sediment matrices [19,20].

We found only two papers using this multivariate analysis combination for assessing air quality [21,22]. In both of them, the algorithms were separately applied to the dataset and then the results were compared and combined for assessing the conclusions.

In this study, we wanted to take advantage of SOM’s capability to extract recurrent variable profiles from the experimental dataset, which usually are one or two orders of magnitude less than those of the input dataset, to obtain a reduced “cleaner” dataset to be used for PMF input. In fact, the reduced dataset obtained by SOM contains less noise and “smoothed” outliers; thus, it is more suitable for further investigation than the original dataset. To the best of our knowledge this is the first time that the abovementioned method has been used to assess air quality and identify possible pollution sources and outliers, such as desert dust events.

To prove the capabilities of this method, we chose a multisite, multiannual dataset containing data from the year 2020, when the COVID-19 pandemic forced governments worldwide to subject their populations to lockdown periods [23]. Several studies have been conducted worldwide to assess the impact of lockdown on air quality. Most of the studies showed that substantial reductions in NO_2_ and NOx were associated with reduced mobility, and thus with reduced amounts of traffic. PM_10_ and PM_2.5_ showed a reduction but with complex signals; more significant reductions were detected in megacities, whereas the decrease was less evident in suburban and rural sites. In some cases, the PM concentration increased [24,25].

## 2. Materials and Methods

### 2.1. Dataset

The hourly data elaborated in this study were collected in the two main cities in the Friuli Venezia-Giulia region (North-East of Italy). One of the cities is by the sea (Trieste—45°39′01″ N 13°46′13″ E, 200,000 inhabitants) and one is inland (Udine—46°04′ N 13°14′ E, 100,000 inhabitants).

The data were retrieved from the Environmental Protection Agency of Friuli Venezia-Giulia Region—Italy (ARPA-FVG) website.

We chose the data collected by monitoring stations classified as “urban traffic” and “city background” stations; thus, four monitoring stations were considered, and they were named according to the city (1 = Trieste, 2 = Udine) and the type (A = “traffic”, B = ”background”). The following pollutants were considered: benzene (Ben), toluene (Tol), PM_10_, NO, and NO_2_. The chosen periods were from 9th of March to 3rd of May from 2018 to 2023.

### 2.2. Data Analysis Method

**Figure 1:**
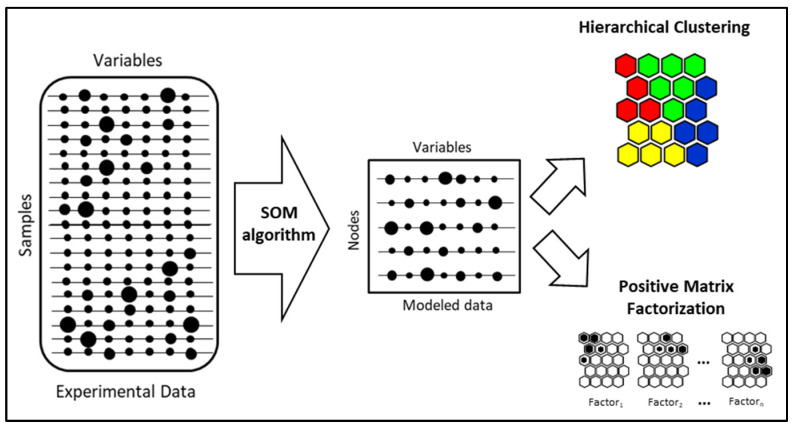
Scheme of data analysis method.

The data were elaborated according to the method shown in Figure 1. First, the SOM algorithm was applied to the dataset, in which each sample is a vector containing a value for each considered variable. The SOM algorithm is a neural network that works in an unsupervised way and it is composed only of the input layer and the output layer, with no hidden layers. The output layer is formed by a list of vectors containing a value for each modeled variable, and each vector is called “node” or “neuron”. Usually, there are ten to one thousand times fewer nodes in proportion to the number of samples, but the nodes can still be used to represent the variability of the input layer. The node values are arranged in a matrix called “codebook”. Each “node” can be represented by a hexagon in a 2D map in which similar nodes are depicted close one to each other, and the evaluation of the multivariate distance between nodes allows us to identify possible clusters [26,27]. Each node represents (i.e., “is similar” in terms of multidimensional Euclidean distance) one or more experimental vectors belonging to the data matrix. Moreover, the way in which the algorithm works reduces the noise of the data [28,29]; this feature is particularly useful when handling instrumental outputs, as was the case in the described study.

In this specific application, the codebook represents recurrent air quality profiles recorded at the sampling sites. The second step was to cluster the nodes applying a hierarchical clustering algorithm using the Euclidean distance and the Ward’s linkage method. In many cases, a clustering method is applied to the codebook [30]. The clustering allows us to obtain “macro-groups” of nodes with similar characteristics, and the benefit of the use of SOM is that the obtained clusters can be depicted on the SOM. The centroid cluster matrix can be thus considered to represent “air quality types” (e.g., “low polluted”, “medium polluted”, highly polluted”, “background”, …).

The centroid matrix can also give some indication about the possible pollution sources, but sometimes the sources can be mixed and there can be no clear interpretation.

The PMF algorithm is a multivariate receptor model that is widely used for identifying source contributions using data collected at the receptor sites [16,31]. We applied the PMF algorithm on the codebook to disentangle the different source contributions, and, by finding the nodes which made greater contributions to the PMF factors, we were able to provide a more complete interpretation of the “air quality types”.

The R software environment was used both for the dataset preparation and the statistical analysis. The data were elaborated using the *SOMEnv* package for the R software environment [32]. The package is based on the *kohonen* package [33,34] for SOM analysis and the *openair* package [35] for managing date/time recordings. The missing data were filled in using the *mdatools* package [36]. The bidimensional HCA plots were obtained using the *pheatmap* package [37]. The EPA PMF (version 5.0 https://www.epa.gov/air-research, accessed on 30 November 2024) was used to perform Positive Matrix Factorization analysis.

## 3. Results and Discussion

### 3.1. Data Cleaning

The data for the four sites were gathered into a single dataset. The dataset was cleaned of all rows containing unavailable values and only the “date–time” combinations present in all four sites were retained. In this way, for each site, we obtained 7999 samples (each sample represents one hour of recording) which were almost evenly distributed in the six years of interest, leading to a dataset containing an overall number of 31,996 rows by five columns (one for each pollutant). The unavailable values were filled in using the algorithm present in the *pca.mvreplace* function contained in the *mdatools* package.

### 3.2. SOM Analysis

**Figure 2:**
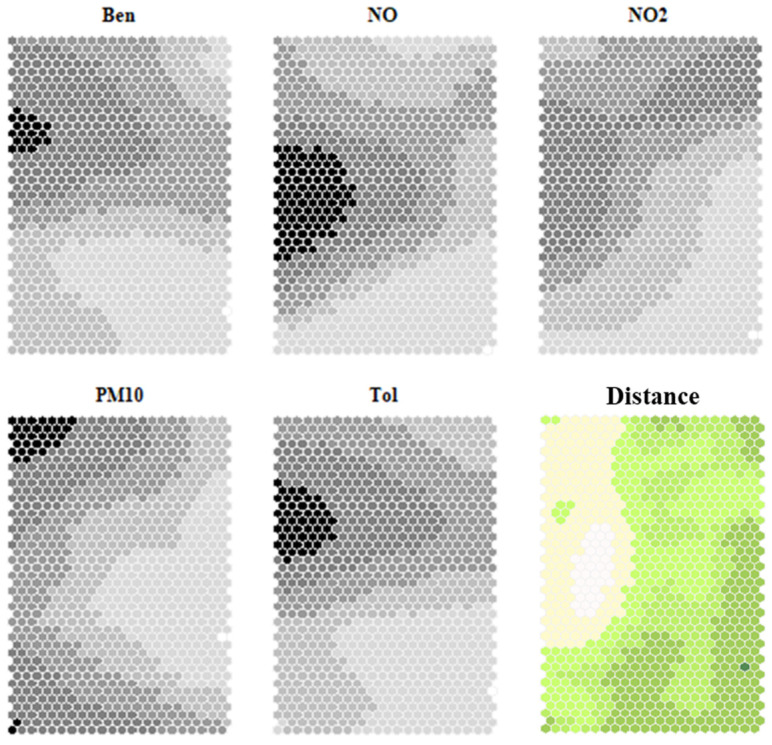
Distribution of the modeled variables on the SOM. The distribution of the single pollutants (Ben, NO, NO_2_, Tol, PM_10_) on each node is depicted in grayscale, from white (lower concentration values) to black (higher concentration values). In the distance map, the distance between a node and its neighbors is depicted with a scale from green to white: the higher the distance, the greater the prevalence of white shading on the scale.

The abovementioned dataset was autoscaled by variable and used to build the SOM model, giving the model no prior knowledge about the site or year classification, to exploit the powerful unsupervised analysis potential of SOM algorithm. The algorithm initialization as well as the number of nodes and map dimensions were selected according to Vesanto’s heuristic rules [26]. Several models were built using different map dimensions and the quality of the model was checked using three well-known parameters: the overall quantization error, the topographic error, and the distribution-matching error [28]. The best model was that with a 41 × 22 map dimension representing 902 recurrent air quality profiles. The obtained map is represented in Figure 2, in which the codebook values are shown in separate maps according to the variable using a grayscale (“heatmaps”). It can be observed that the highest pollutant values are depicted on the top and left edges of the map. In contrast, the lowest values are depicted in the bottom-right area of the map. As a rule, similar nodes are depicted close together on the map to maintain the dataset topology, but there can be some edges among nodes. The edges allow us to visualize possible node grouping and are usually represented using the distances between neighborhood nodes. The distance map represented in Figure 2 shows that there is a discontinuity (white “peak” area) spreading from the upper left part toward the map center. This is in accordance with the behavior observed in the heatmaps.

### 3.3. Hierarchical Clustering

**Figure 3:**
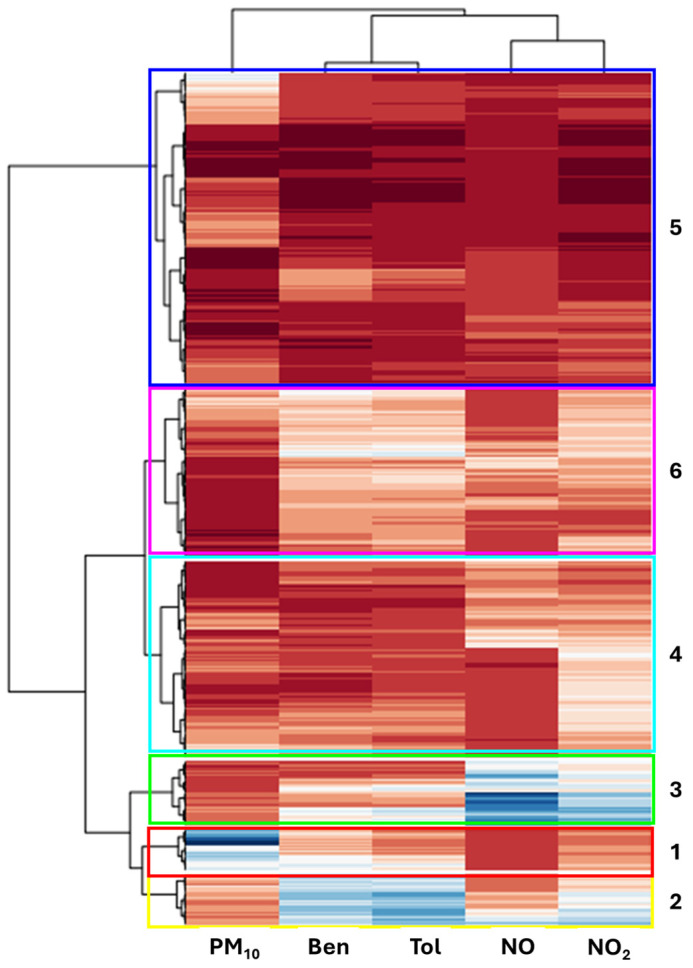
Clustered two-way HCA map. Each row represents a node, while each column represents the values of the modeled variables retaining the autoscaling operated before SOM analysis; thus, the color scale represents low (dark red) to high (dark blue) values. The six clusters obtained are depicted by rectangles and the assigned cluster number is indicated on the right-hand side of the figure.

The nodes were then grouped using hierarchical clustering for a more in-depth and quantitative analysis, as stated in Section 2.2. The clustering algorithm was applied to the codebook. The clustering is shown in Figure 3 in a two-way mode in which both the nodes (arranged in rows) and the modeled variables (arranged in columns) were grouped. We evaluated the quality of clustering from 2 to 10 clusters using the Davies–Bouldin index [38], which is regarded as a robust cluster validity index [39]. The best number of clusters was six. The six clusters are highlighted by colored rectangles in Figure 3; moreover, the rectangles show where the HCA “branches” were cut row-wise in order to obtain the six clusters.

**Figure 4:**
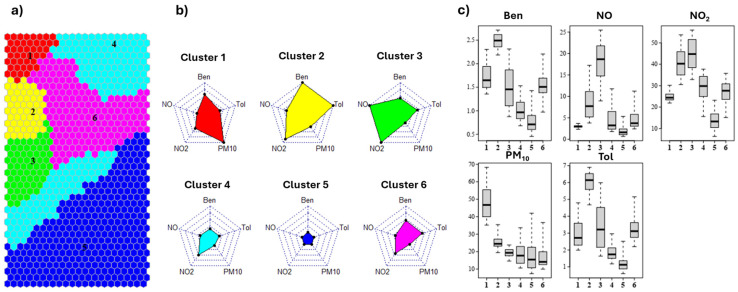
(**a**) Division of SOM nodes into 6 clusters as obtained by HCA; (**b**) representation of the cluster centroid values by radar plots; (**c**) distribution of the modeled values for each cluster, as defined by SOM. For this figure, we used the same cluster color code as the one used in Figure 3.

In Figure 4, the grouping on the map is shown together with the “air type” variable profiles represented by radar plots. The boxplots on the right show the spread of the modeled values for each cluster. The node clusters provide insights into the characteristics present in the pollutants data. It can be observed that clusters 1, 2, and 3 (highlighted in the top-left part of the map) represent “highly polluted” air with relatively high values of PM_10_ (cluster 1), Ben and Tol (cluster 2), NO and NO_2_ (cluster 3), compared to the others. By observing the boxplot, it can be noticed that for cluster 3, the variable values are fairly spread out, with the exception of PM_10_. Clusters 1, 2, and 3 are in the same map area outlined by the distance discontinuity depicted in Figure 2. Moreover, the distribution of the variable values of the same area can be identified in the heatmaps presented in Figure 2.

Cluster 5 occupies the entire bottom-right side of the map, as it contains many nodes with the smallest concentrations of pollutants among all other clusters. It can be associated with the “background” air type. A so-called “background” pollutant concentration is the lowest level of concentration that can be reached in an area, when there are no “active” sources nearby; only a level of concentration that can be attributed to long-distance transport and aged pollutant distribution in the area. Cluster 6 and 4 can be classified as “medium” and “low” polluted air, respectively.

The significance of the difference between the clusters was assessed using a Kruskal–Wallis non-parametric test. *p*-values of <0.001 were obtained for all the variables. Then, the Wilcoxon test with Bonferroni correction was used to assess the paired difference between clusters. Almost all the paired differences were significant; the results are reported in detail in the Supplementary Material.

**Figure 5:**
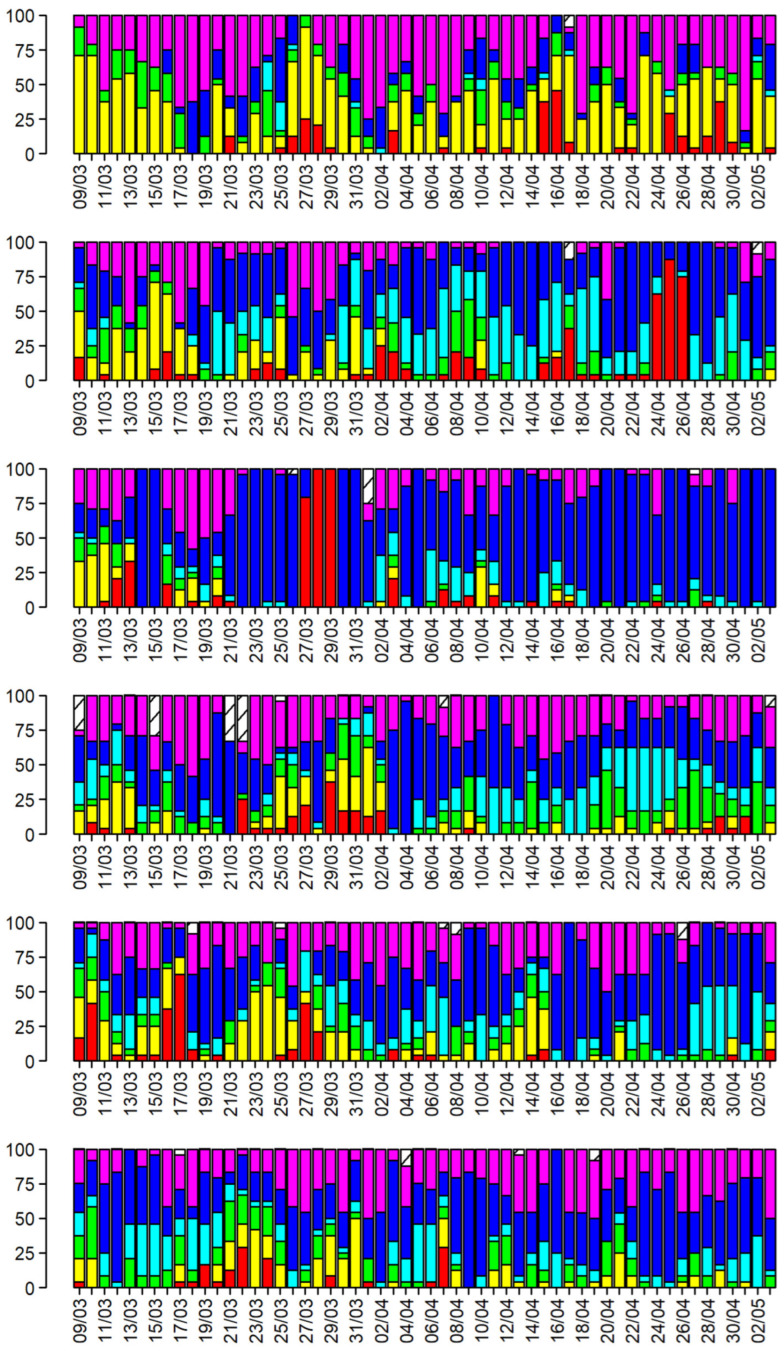
Barplots representing the daily percentage distribution of clusters for site A1. From the top to the bottom of the figure: years from 2018 to 2023. For this figure, we have used the same cluster color code as the one in Figure 4.

The obtained clusters can also be linked to the different monitoring stations and the timeframe in which the pollutant levels were recorded. This evaluation was performed for all stations by labeling the percentage of each cluster for each day, according to the monitoring station and year. In Figure 5, the stacked daily bar plots for site A1 are represented, with the same color code provided for the SOM in Figure 4. The plots for all the sites are reported in the Supplementary Material.

Figure 5 shows the effect of the lockdown in 2020, with high prevalence of cluster 5, showing that the impact of pollutants was low for most of this period, according to results highlighted in other studies [23]. This effect can also be observed at sites B1 and A2 and, to a lesser extent, at B2. Thus, the effect is more evident in “traffic”-monitoring stations (A1 and B1), in accordance with other studies [24,25].

It can also be seen that, for the year 2020 alone, both A- and B-type sites in the respective cities show similar behavior, smoothing out the differences that can be observed in other years.

Cluster 2 seems to be a peculiar “air type” of site 1, characterized by the presence of high values of Ben and Tol, and partly of NO_2_. It can be related to two possible sources. The first source, until its closure in 2020, was an integrated-cycle steel plant which, in particular, released benzene from its coke distillation ovens [40,41]. The second source, that is still active, is the presence of a harbor with a petrochemical terminal, which can release several types of hydrocarbons during unloading operations [42]. The presence of NO_2_ can be related to the combustion process for the first source and to ship stack emissions for the second one.

Cluster 3 shows the highest values of NO, which is a product of primary combustion. After the emission, it is oxidized to NO_2_ in tens of minutes, depending on the presence of oxidants, such as ozone, in the air [43,44]. From the daily plots, it can be observed that cluster 3 is barely visible for 2020 for all of the sites, confirming that the primary combustion source close to the monitoring stations (i.e., traffic) was largely absent, as other sources of combustion producing NO (e.g., domestic heating) that are not so close to the stations exploit their effect in NO_2_ concentrations.

For 2019 and 2020, some days (at the end of March and at the end of April, respectively) show a high percentage of cluster 1, which is characterized by a high level of PM_10_.

The SOM output allows us to detect possible outliers observing the values of the “so-called” quantization errors (QEs) [45,46].

A quantization error is the multidimensional distance of a sample from the node that best represents it (Best Matching Unit). A relatively high value of QEs means that the sample could be a possible outlier. The sample outliers were explored and reported in the Supplementary Material in stacked plots split by site and year; in 2020, all the sites showed outliers belonging to cluster 1. To a minor extent, such outliers were also present in 2019, although none were observed for site B2. The outliers correspond to two intense desert dust intrusions in the Northern Adriatic Sea area [47].

In site B2, in 2023, we noted the presence of samples with high QEs at the end of March for few hours. They could be related to a possible transient event, such as an accidental gasoline spill or road asphalting operation.

### 3.4. Positive-Matrix Factorization

**Figure 6:**
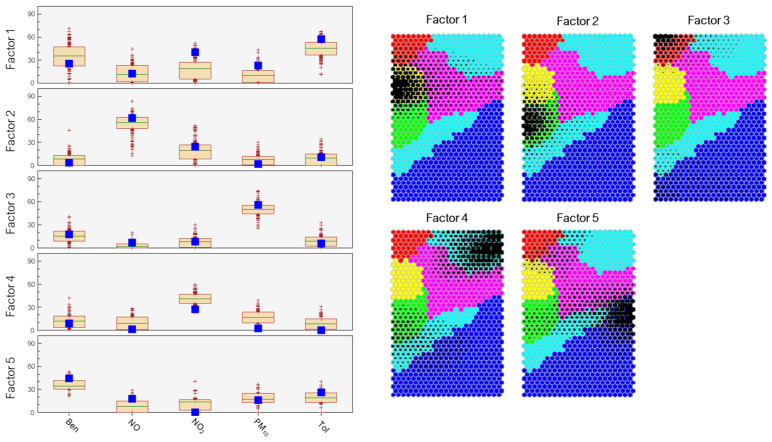
On the left: Variability in the % contribution of each species to the respective PMF factor (sum of factors  =  100%). The base run is shown as a blue box for reference. On the right: the nodes that made greater contributions to a factor are represented in black, with a greater amount of black shading indicating a more substantial contribution.

The codebook was used as input for the PMF algorithm and the uncertainties used for the variables were 6.2% for NO and NO_2_, 10.9% for Ben and Tol, and 10% for PM_10_, respectively. Considering the abovementioned clustering results, we tried to identify four to six emission sources/factors, obtaining better model quality parameters for five factors. The bootstrap method was employed to mitigate uncertainty and validate the precision of the PMF model. The first three factors were perfectly replicated in over 100% of the runs; factor 4 in over 87% of the runs, and factor 5 in over 42% of the runs. No runs were left unmapped, indicating that bootstrap uncertainties are interpretable and the number of factors may be suitable. For all factors, 80% of the species from the base run fell within the interquartile range (25th–75th percentile) of the bootstrap runs, thereby underscoring the robustness of the PMF modeling. The five factor profiles are shown in Figure 6 along with the nodes of the map, which mainly contributed to the factors. The factor fingerprints are reported in the Supplementary Material.

Factor 1 mainly shows an hydrocarbon source with a partial contribution of NO_2_. Most of the nodes that contribute to this factor are those in cluster 2, which has already been recognized as a peculiarity of site 1. This is a mixed industrial source, as explained in detail in Section 3.3. Factor 2 shows the highest percentage of NO, indicating a primary source of combustion that, considering the monitoring station positioning described at Section 2.1, is mainly related to road transport. The nodes that mostly contribute to this factor are those belonging to cluster 3, which was described in detail in Section 3.3.

Factor 3 contains the highest percentage of PM_10_, with nearly no percentage of the other pollutants indicating a “pure” dust source. The nodes with the greatest contribution to this factor are those belonging to cluster 1. In this cluster, the outliers represent the desert dust intrusion were found, as described in detail in Section 3.3.

Factor 4 indicates an aged combustion source, with the presence of NO_2_ and PM_10_. The nodes with the greatest contribution to this factor are those belonging to cluster 4, which represented “low polluted” air; thus, the results are consistent. Factor 5 indicates a mixed source of PM_10_ and hydrocarbons. The nodes with the greatest contribution to this factor are fairly spread out on the SOM, with some of them gathered in a sub-area of cluster 5; moreover, this was the factor with the lowest precision. Thus, there is no straightforward interpretation of this factor.

The nodes belonging to cluster 6 did not show a clear contribution to a specific factor; thus, they probably originate from a combination of sources, in accordance with the cluster interpretation of “medium-level” pollution.

Using PMF, we recognized and confirmed the source nature of four out of six clusters identified by HCA, allowing a more in-depth interpretation of the spatial and temporal air quality variation in the two monitored cities.

## 4. Conclusions

In this study, we used a combined approach of multivariate analysis involving SOM, HCA, and PMF to disentangle multiannual, multisite data in a single elaboration without previously separating the sites and years. The approach proved to be valid, allowing us to detect the site peculiarities in terms of pollutant sources, the variation in pollutant profiles during years, and the outliers, affording a reliable interpretation. In detail, SOM allowed us to obtain recurrent pollutant profiles while retaining the relevant information and reducing the noise. HCA allowed us to classify different air types and evaluate their impact on the population. PMF was used to recognize and confirm the pollutant sources identified by HCA.

## Data Availability

Data available on request.

## Supplementary Materials

The following supporting information can be downloaded at https://www.mdpi.com/article/10.3390/toxics13020137/s1, Table S1: Wilcoxon test results; Figure S1: Barplots representing the daily percentage distribution of clusters for site A1. From the top to the bottom of the figure: years from 2018 to 2023; Figure S2: Barplots representing the daily percentage distribution of clusters for site B1. From the top to the bottom of the figure: years from 2018 to 2023; Figure S3: Barplots representing the daily percentage distribution of clusters for site A2. From the top to the bottom of the figure: years from 2018 to 2023; Figure S4: Barplots representing the daily percentage distribution of clusters for site B2. From the top to the bottom of the figure: years from 2018 to 2023; Figure S5: Quantization error plots for site A1. From the top to the bottom of the figure: years from 2018 to 2023; Figure S6: Quantization error plots for site B1. From the top to the bottom of the figure: years from 2018 to 2023; Figure S7: Quantization error plots for site A2. From the top to the bottom of the figure: years from 2018 to 2023; Figure S8: Quantization error plots for site B2. From the top to the bottom of the figure: years from 2018 to 2023; Figure S9: PMF results: Factor fingerprints.
