# Supplementary material for: Disentangling Multiannual Air Quality Profiles Aided by Self-Organizing Map and Positive Matrix Factorization
Source: Toxics. 2025 Feb 14;13(2):137. doi: 10.3390/toxics13020137 (PMC11860770; doi:10.3390/toxics13020137)
Supplement: Supplementary file 1 [file toxics-13-00137-s001.zip › toxics-3430674-supplementary.pdf]

SUPPLEMENTARY MATERIAL

Disentangling multiannual air quality profiles aided by Self-Organizing Map and Positive Matrix Factorization

Stefano Fornasaro, Aleksander Astel, Pierluigi Barbieri and Sabina Licen

- Table S1: Wilcoxon test results
- Figure S1: Barplots representing the daily percentage distribution of clusters for site A1. From the top to the bottom of the figure: years from 2018 to 2023
- Figure S2: Barplots representing the daily percentage distribution of clusters for site B1. From the top to the bottom of the figure: years from 2018 to 2023
- Figure S3: Barplots representing the daily percentage distribution of clusters for site A2. From the top to the bottom of the figure: years from 2018 to 2023
- Figure S4: Barplots representing the daily percentage distribution of clusters for site B2. From the top to the bottom of the figure: years from 2018 to 2023
- Figure S5: Quantization error plots for site A1. From the top to the bottom of the figure: years from 2018 to 2023
- Figure S6: Quantization error plots for site B1. From the top to the bottom of the figure: years from 2018 to 2023
- Figure S7: Quantization error plots for site A2. From the top to the bottom of the figure: years from 2018 to 2023
- Figure S8: Quantization error plots for site B2. From the top to the bottom of the figure: years from 2018 to 2023
- Figure S9: PMF results: Factor fingerprints

Table S1: Wilcoxon test results (p-value)

**Cluster Number**	**Cluster Number**	**Benzene**	**NO**	**NO_2**	**PM_{10}**	**Toluene**
2	1	< 0.001	< 0.001	< 0.001	< 0.001	< 0.001
3	1	< 0.05	< 0.001	< 0.001	< 0.001	N.S.
4	1	< 0.001	N.S.	< 0.001	< 0.001	< 0.001
5	1	< 0.001	< 0.001	< 0.001	< 0.001	< 0.001
6	1	< 0.01	< 0.001	< 0.01	< 0.001	< 0.05
3	2	< 0.001	< 0.001	< 0.05	< 0.001	< 0.001
4	2	< 0.001	< 0.001	< 0.001	< 0.001	< 0.001
5	2	< 0.001	< 0.001	< 0.001	< 0.001	< 0.001
6	2	< 0.001	< 0.001	< 0.001	< 0.001	< 0.001
4	3	< 0.001	< 0.001	< 0.001	N.S.	< 0.001
5	3	< 0.001	< 0.001	< 0.001	< 0.01	< 0.001
6	3	N.S.	< 0.001	< 0.001	< 0.001	N.S.
5	4	< 0.001	< 0.001	< 0.001	< 0.01	< 0.001
6	4	< 0.001	N.S.	< 0.001	< 0.05	< 0.001
6	5	< 0.001	< 0.001	< 0.001	N.S.	< 0.001

N.S. = Not Significant

A1

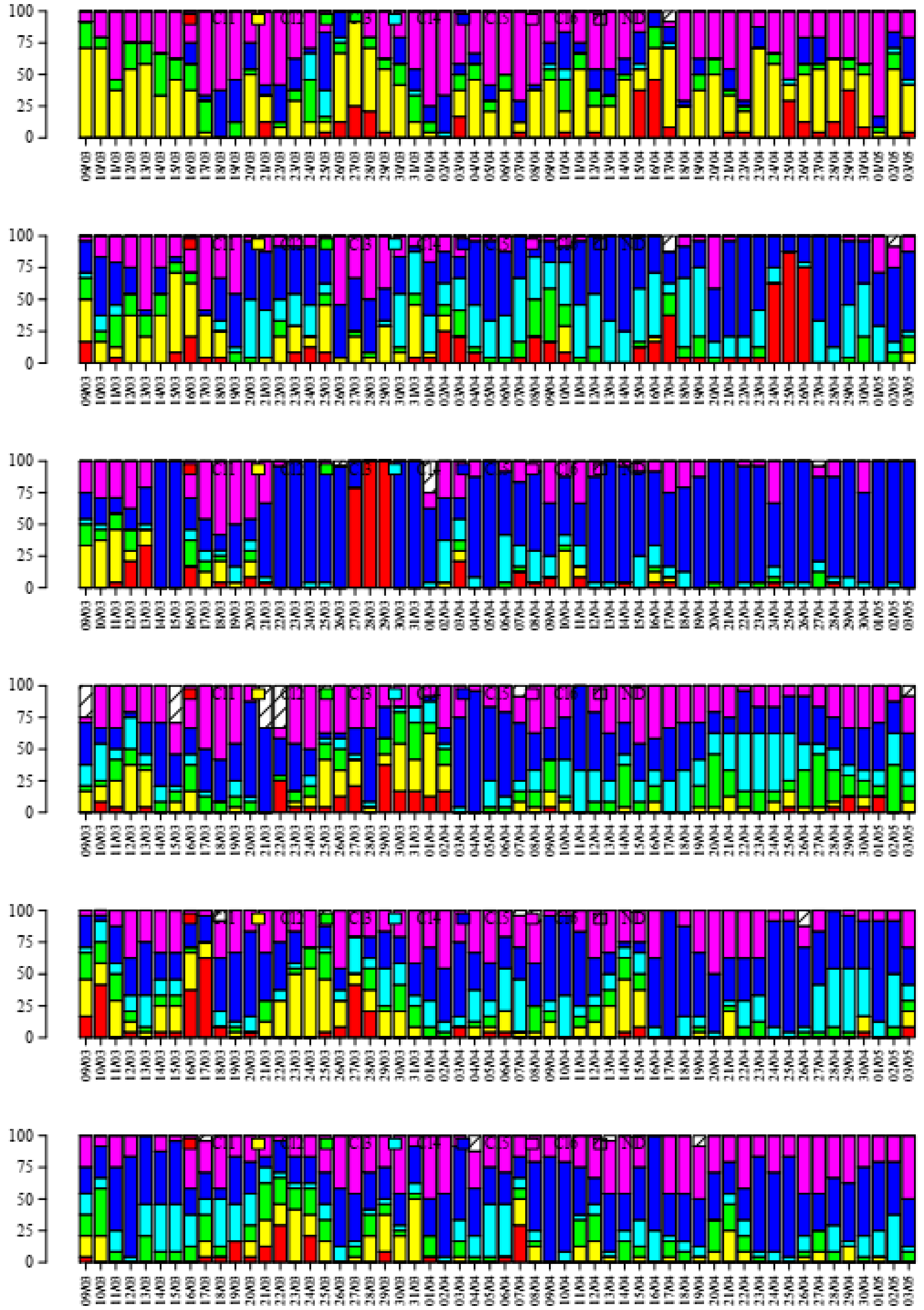

Figure S1: Barplots representing the daily percentage distribution of clusters for site A1. From the top to the bottom of the figure: years from 2018 to 2023

B1

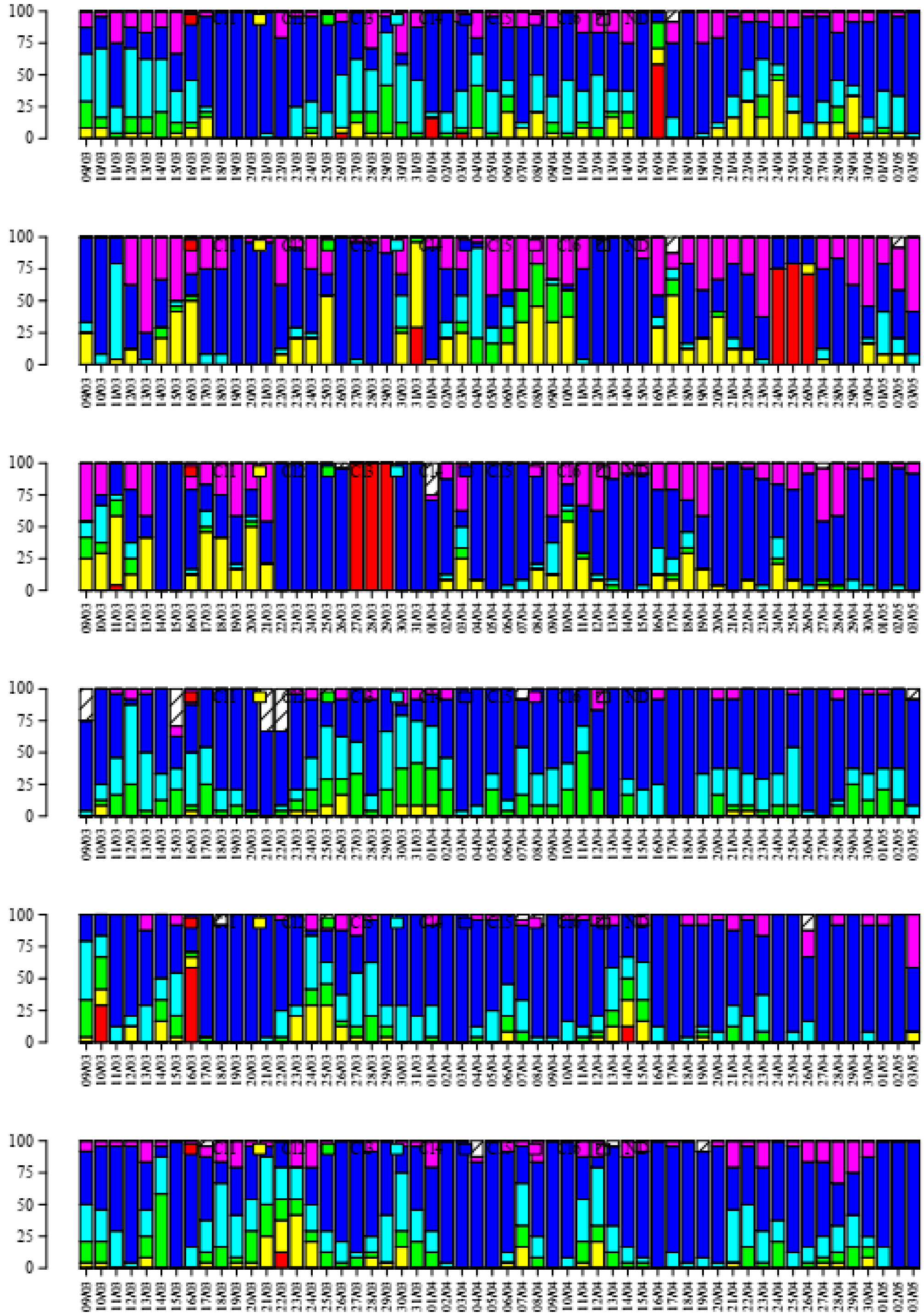

Figure S2: Barplots representing the daily percentage distribution of clusters for site B1. From the top to the bottom of the figure: years from 2018 to 2023

A2

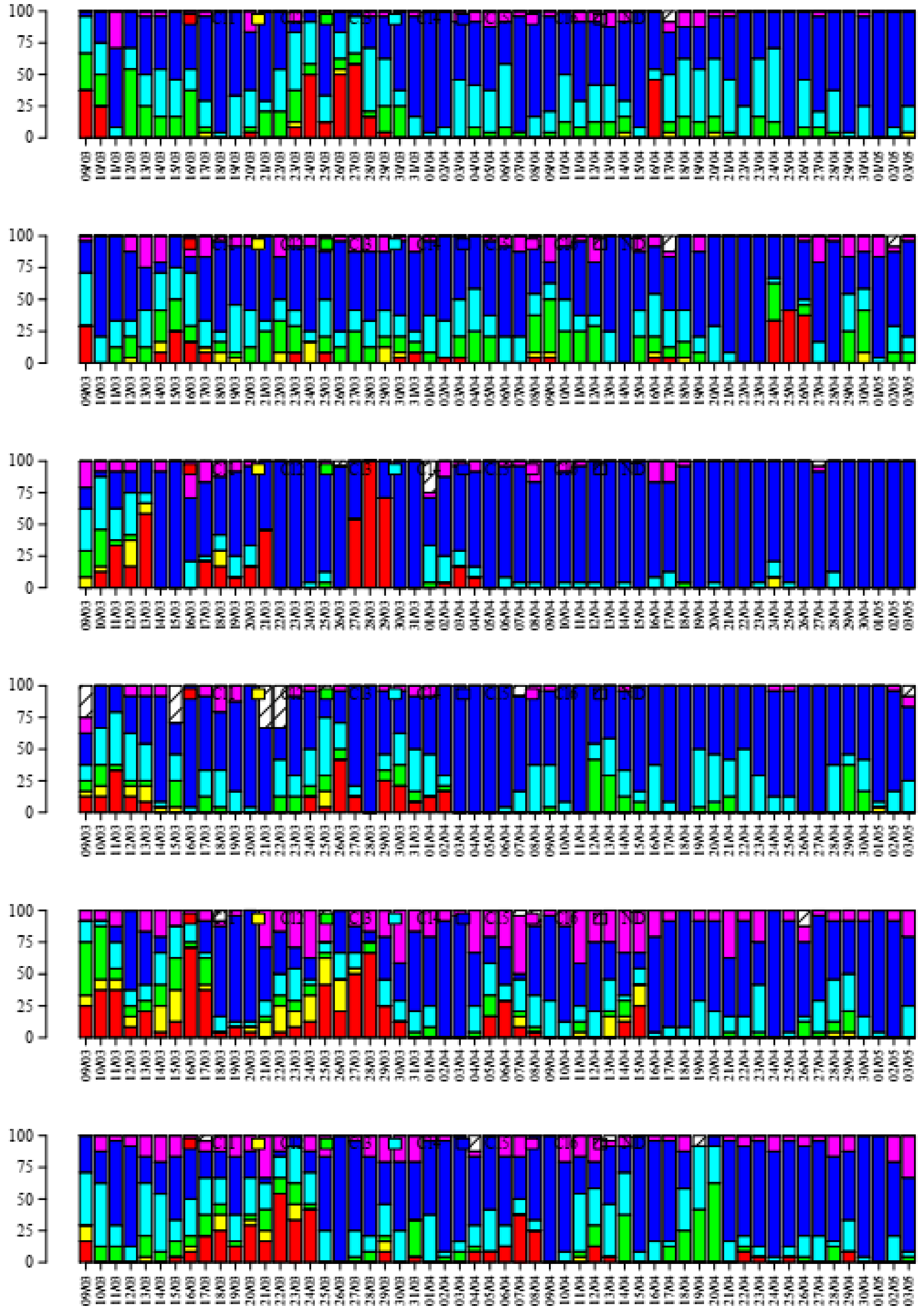

Figure S3: Barplots representing the daily percentage distribution of clusters for site A2. From the top to the bottom of the figure: years from 2018 to 2023

B2

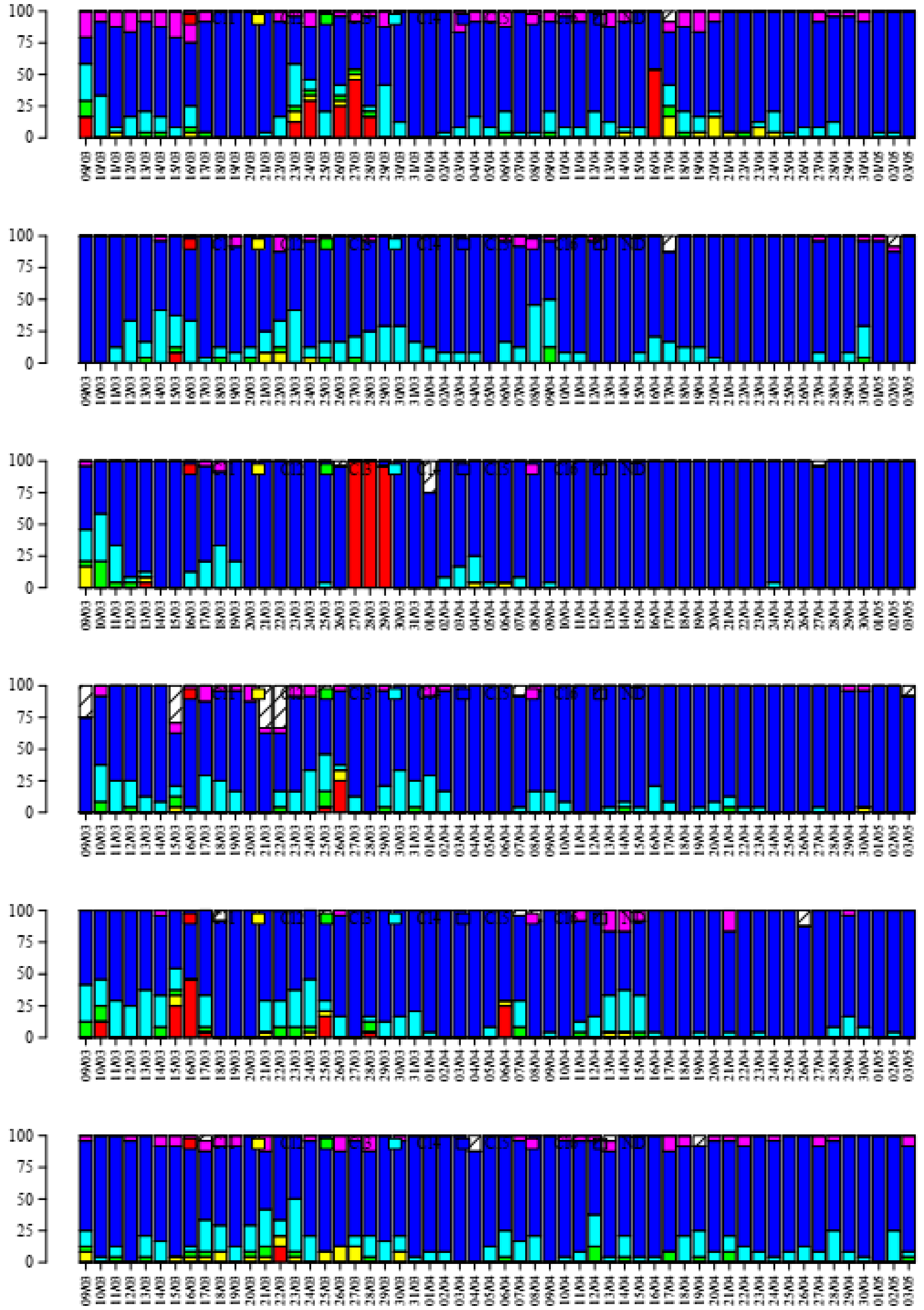

Figure S4: Barplots representing the daily percentage distribution of clusters for site B2. From the top to the bottom of the figure: years from 2018 to 2023

A1

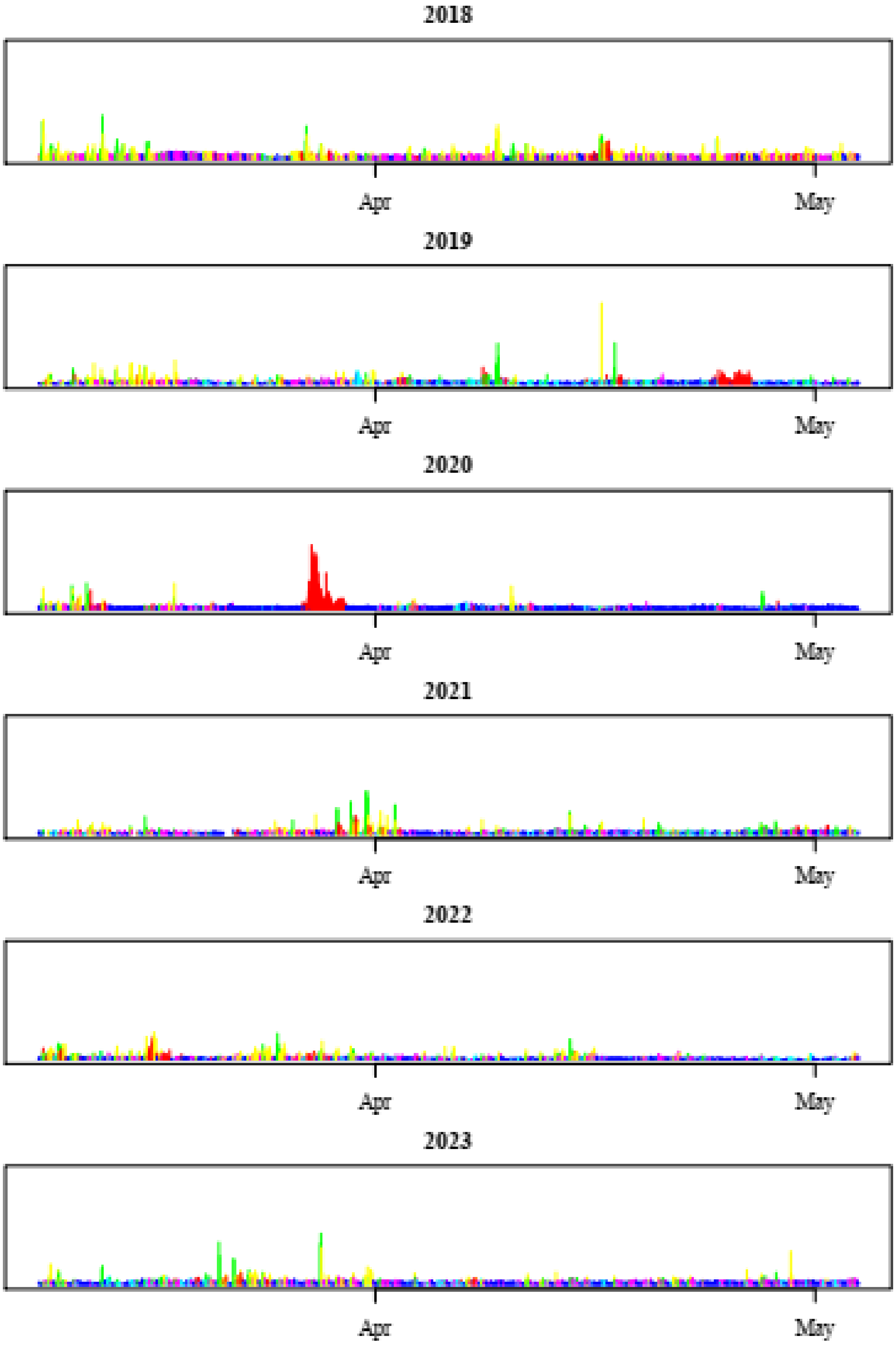

Figure S5: Quantization error plots for site A1. From the top to the bottom of the figure: years from 2018 to 2023

B1

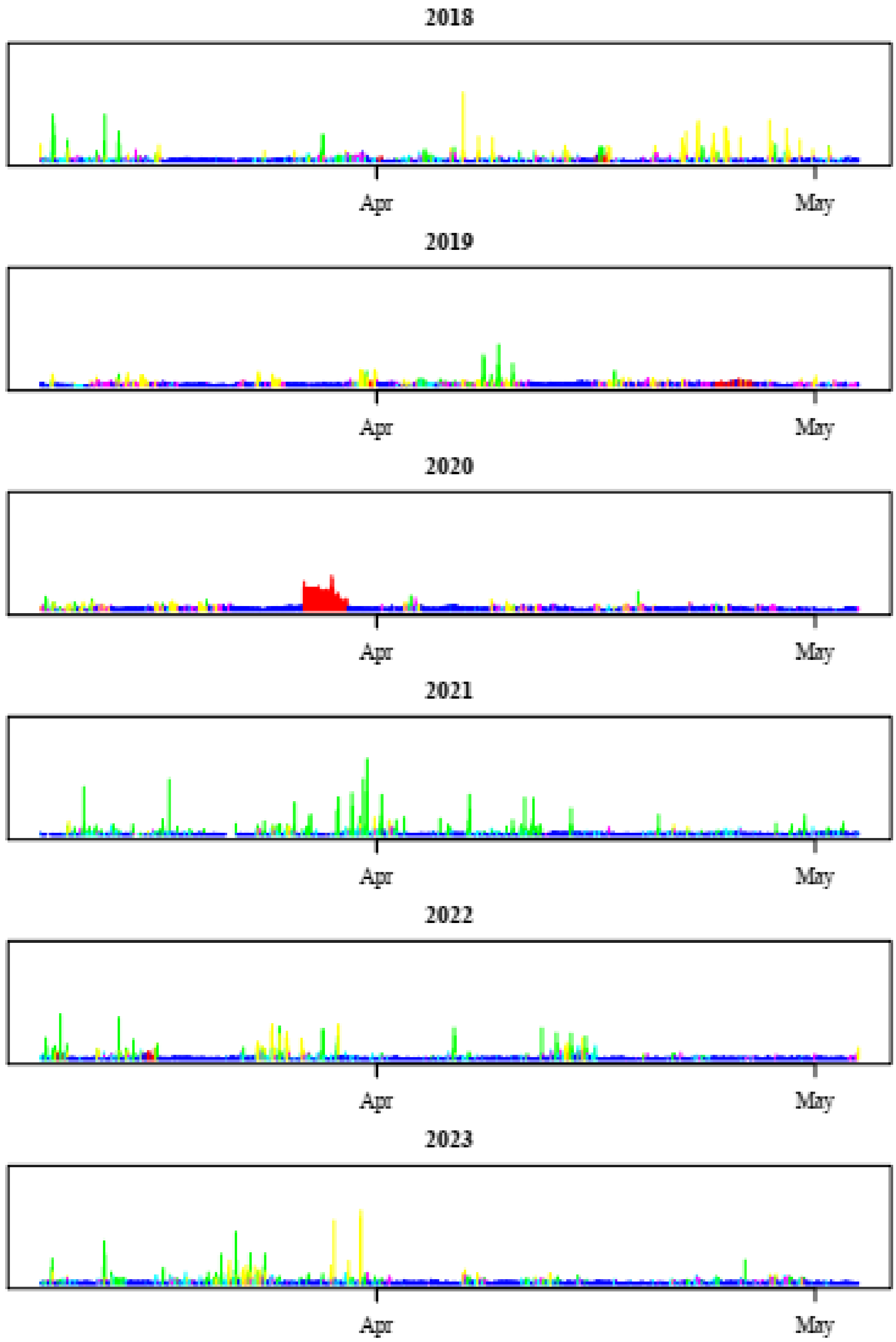

Figure S6: Quantization error plots for site B1. From the top to the bottom of the figure: years from 2018 to 2023

A2

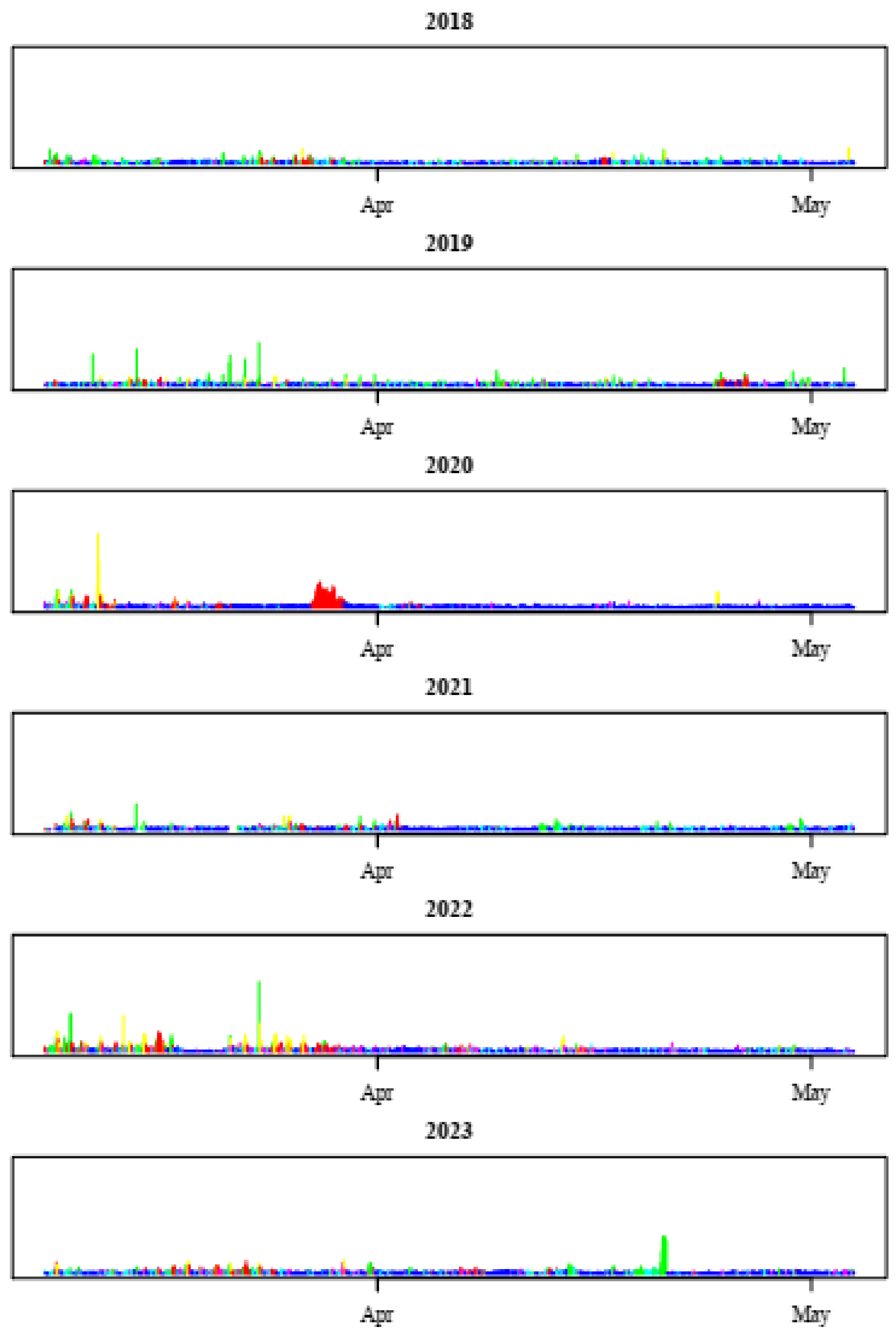

Figure S7: Quantization error plots for site A2. From the top to the bottom of the figure: years from 2018 to 2023

B2

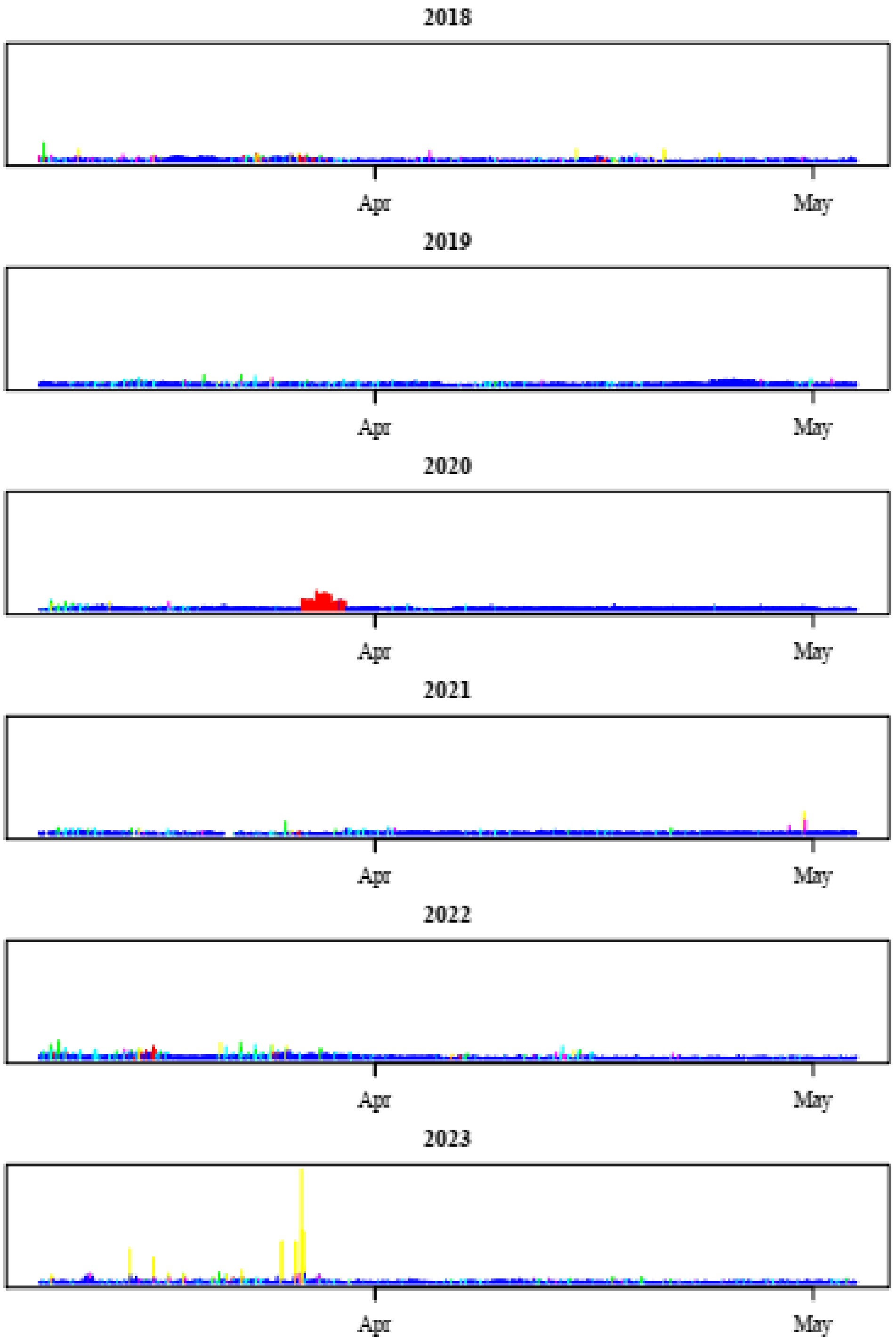

Figure S8: Quantization error plots for site B2. From the top to the bottom of the figure: years from 2018 to 2023

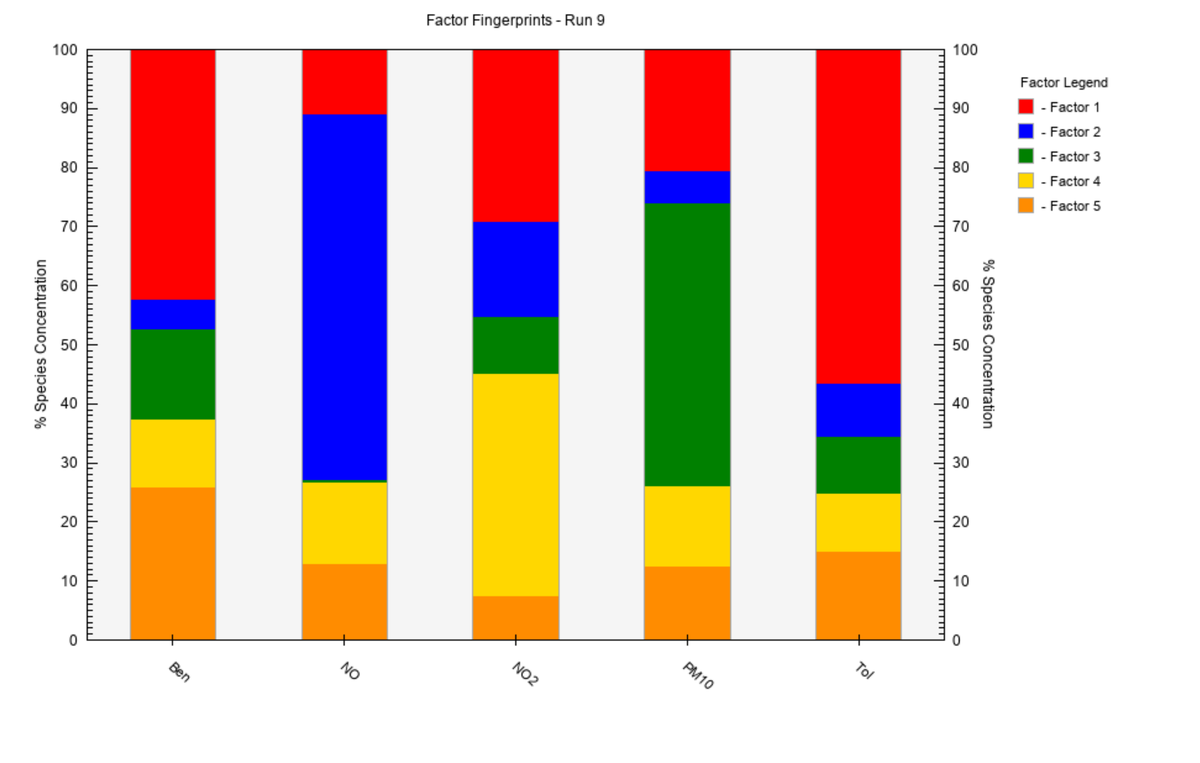

Figure S9: PMF results: Factor fingerprints
